# Cognition and resective surgery for diffuse infiltrative glioma: an overview

**DOI:** 10.1007/s11060-012-0811-x

**Published:** 2012-02-24

**Authors:** Martin Klein, Hugues Duffau, Philip C. De Witt Hamer

**Affiliations:** 1 Department of Medical Psychology, VU University Medical Center, Van der Boechorststraat 7, 1081 BT Amsterdam, The Netherlands; 2 Department of Neurosurgery, Hôpital Gui de Chauliac, and INSERM U1051, Institute for Neurosciences of Montpellier, Team “Brain Plasticity, Human Stem Cells and Glial Tumors,” Montpellier University Medical Center, Montpellier, France; 3 Neurosurgical Center Amsterdam, VU University Medical Center, Amsterdam, The Netherlands

**Keywords:** Glioma, Cognitive function, Neurosurgery, Radiotherapy, Epilepsy, Medical treatment

## Abstract

Compared to classical oncological outcome measures such as time to progression and survival, the importance of cognitive functioning in patients with diffuse infiltrative brain tumors has only recently been recognized. Apart from the relatively low incidence and the invariably fatal outcome of gliomas, the general assumption that cognitive assessment is time-consuming and burdensome contributes to this notion. Our understanding of the effects of brain surgery on cognition, for instance, is largely based on studies in surgical patients with refractory epilepsy, with only a limited number of studies in surgical patients with gliomas. The impact of other factors affecting cognition in glioma patients such as direct tumor effects, radiotherapy and chemotherapy, and medical treatment, including anti-epileptic drugs and steroids, have been studied more extensively. The purpose of this paper is to provide an overview of cognition in patients with diffuse infiltrative gliomas and the impact of resective surgery as well as other tumor and treatment-related factors.

## Introduction

Although an increasing number of studies indicate that primary brain tumors and their treatment are often associated with cognitive deficits, there is still limited knowledge about their incidence, nature, severity, and causes. Since patients with diffuse infiltrative gliomas (WHO grade 2–4) cannot be cured, palliation of symptoms and maintenance or improvement of physical functioning and health-related quality of life (HRQOL) are important goals of treatment. Evaluation of treatment in these patients should thus not only focus on (progression-free) survival, but should also aim at functional outcome and at adverse treatment effects on the normal brain. Functional outcome refers to neurological, cognitive, professional, and social performance of an individual, usually abstracted as HRQOL. With regard to the effects of tumor and treatment on the normal brain, cognitive functioning is a useful outcome measure for brain tumor patients, since cognitive deficits, even mild, may negatively affect HRQOL [1], professional reintegration, interpersonal relationships, and leisure activities.

Many potential factors contribute to cognitive functioning. In attempting to determine the isolated effect of resective surgery on cognition, the multifactorial processes involved should be recognized. These factors include premorbid level of cognitive functioning, distant mechanical effects on the normal brain by the lesion, epilepsy, medication, and other oncological treatments.

Cognitive functioning and HRQOL assessment are used as secondary outcome measures in several clinical trials and can also serve as an early indicator of disease progression and have prognostic significance [2, 3], thereby providing additional arguments in clinical decision making. To illustrate the clinical decision making process, a typical patient is described. This case description allows us to describe the patterns of cognitive functioning related to tumor and/or treatment within an ecological context, stressing the importance of conceptualizing patients’ cognitive deficits, their families, and their caregivers as an integrated “system” with all the humanistic and ethical aspects that entails, rather than as cognitive deficits in isolation.

**Fig. 1:**
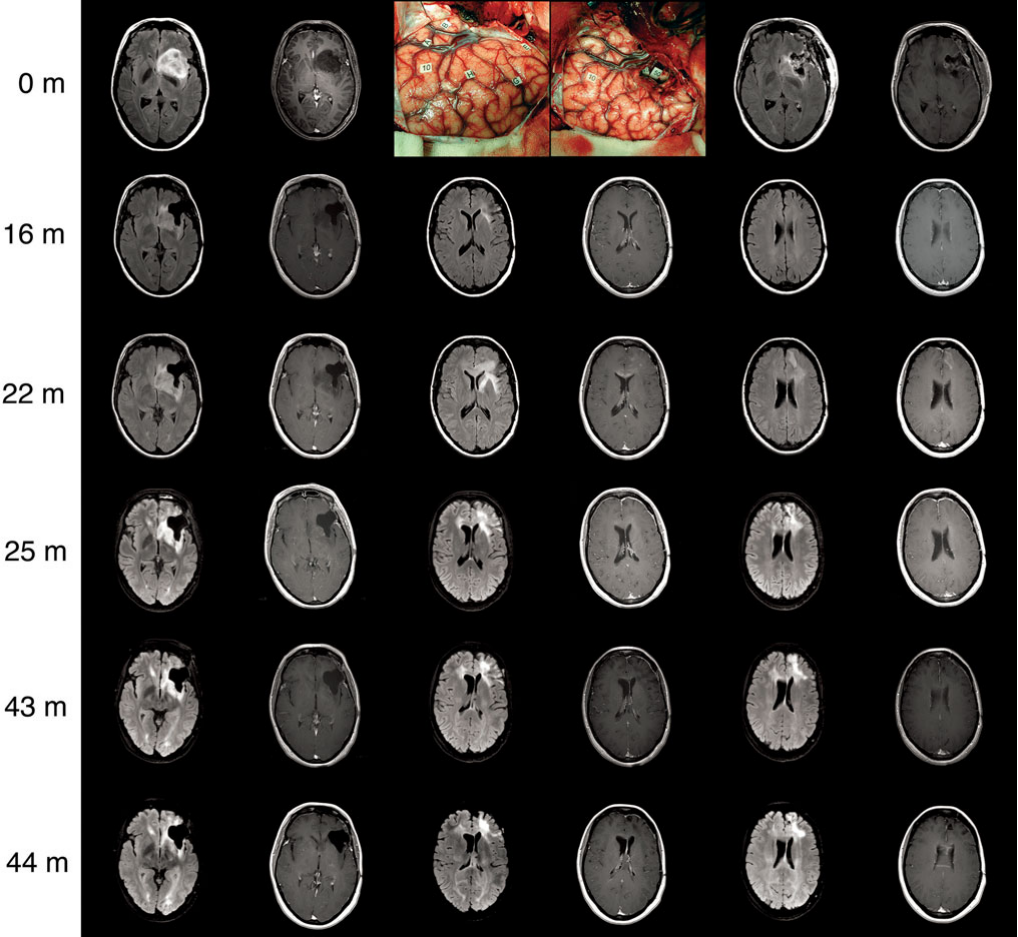
Rapid transformation from a WHO grade II oligodendroglioma at the time of surgery into an anaplastic oligodendroglioma at 22 months

A 36-year old female presented with several elementary seizures in the 3 months preceding presentation, characterized by foul smell followed by inattention and dysphasia. Otherwise she suffered from fatigue for 10 years which was diagnosed as ‘chronic fatigue syndrome’. She was married, had one child and worked as an administrative employee in an international organization. She had a normal neurological examination and was right-handed. The first MRI showed a T2/FLAIR hyperintense lesion of 50 ml anterior in the left insula with no enhancement after gadolinium suggesting a low-grade glioma (Fig. 1a). She started on carbamazepine 200 mg bid. Information was provided on resective surgery with its presumed beneficial impact on time to progression and survival, the acceptable low risk of permanent neurological deficits when using brain mapping under local anesthesia, and the unknown risk of cognitive decline, as well as alternative treatment options consisting of a biopsy and radiotherapy or chemotherapy and radiological follow-up with delayed treatment. She was highly motivated to undergo resective surgery with language mapping. A baseline assessment for language and neuropsychological examination was obtained showing a score on the Boston naming test in the lower normal range.

### Tumor and surgery effects

Apart from seizures, as presented by the patient in our case description, brain tumor patients may present with headaches, focal neurologic signs, and cognitive impairment. Cognitive deficits associated with brain tumors can be induced by compression of normal brain either directly or indirectly by reactive edema. Reduction of compression has not only been shown to improve cognitive function after removal of non-invasive lesions such as meningiomas [4], associated with improvement in attentional functioning, or arachnoidal cysts [5], associated with better dichotic perception and overall memory performance in patients but even after cranioplasty [6], where post surgery patients had improved language and reasoning abilities. Apart from compression, the invasion of parenchymal glial tumors directly into functional brain regions or indirectly by disconnection of structures can further contribute to cognitive deficits [7–9].

After radiological diagnosis, resective surgery is usually the first of several treatment modalities for patients with brain tumors. Resective surgery aims to balance functional outcome (e.g., minimization of neurological deficits) with oncological outcome (e.g., maximization of tumor removal) to improve survival. A vast body of literature exists on the impact of surgery for a variety of brain lesions on neurological outcome, such as motor strength and language. However, due to the limited number of studies including pre- and postoperative cognitive evaluations, the true incidence and extent of cognitive dysfunction specifically related to resective brain tumor surgery is unknown.

The patient had a resection in which the brain structures involved in language determined the boundaries of resection (Fig. 1b, c). A subtotal resection of 92% (4 ml residual tumor) was obtained (Fig. 1d). After surgery in the first week a mild dysnomia completely resolved, and her fatigue was increased for 2 months. The pathology was oligodendroglioma WHO grade 2. No adjuvant treatment was advised and she had radiological follow-up every 6 months.

Cognitive outcome after resective surgery for temporal lobe epilepsy and for brain tumors will be discussed, respectively.

Studies in patients with non tumor-related intractable epilepsy showed cognitive improvement (e.g., memory or verbal fluency) with adequate seizure control after temporal resections [10–16]. Less extensive resection of the mesiotemporal structures seems to correlate with better memory outcome compared with more extensive temporal lobectomy according to some groups [17–19], whereas others have reported conflicting observations [20–22]. Furthermore, dominant temporal lobe resections have been correlated with verbal memory decline in a subset of patients [23–29], whereas non-dominant temporal lobe resections were correlated with visuospatial memory decline [29–35].

Studies of extra-temporal resective surgery for intractable epilepsy also yielded variable cognitive outcomes. After unilateral removal of frontal cortex cognition was either unchanged [36, 37], or specific cognitive domains were impaired, such as reaction time [38, 39], impulsivity [40], advance information utilization [41], conditional learning [42], or search and retrieval strategies [43]. Furthermore, identification of faces and categorization of emotional facial expression was impaired after either frontal or temporal cortex resection [44]. Olfactory identification was impaired following unilateral excision of the temporal lobe or the orbitofrontal cortex on either side [45].

Cognitive outcome has not been systematically assessed for resective brain surgery in patients with brain tumors, although several interesting observations have been done in smaller observational cohort studies.

Firstly, cognitive improvement has been observed in several studies after brain tumor resection. Long-term improvement of verbal memory compared to preoperative assessment has been reported after low-grade glioma resections in frontal premotor and anterior temporal areas [46–48], usually after a transient immediate postoperative worsening. Additionally, regardless the precise tumor location, patients with low-grade gliomas in the right hemisphere run a lower risk of developing cognitive deficits after surgery [49]. Cognitive improvement has also been observed after surgical resection of high-grade gliomas [50], specifically in word fluency, verbal memory, and visuospatial memory. However, one study also suggests that tumor histology might not be that important in the prediction of cognitive outcome following surgery [49].

Secondly, in some studies stable cognitive performance was observed after brain tumor resection. For instance, patients with tumors of the third ventricle demonstrated cognitive impairment in memory, executive functioning, and fine manual speed prior to surgery, without worsening of cognition after surgical removal [51, 52]. Out of several executive tasks, only letter fluency performance was impaired in patients after glioma surgery in left frontal locations compared with right frontal and posterior lesions [53]. Visuospatial processing in patients after resective glioma surgery in left and right, frontal and parietal locations was comparable to that of normal subjects according to one study [54] and impaired spatial and positional memory processing was demonstrated in patients with tumors in the right posterior parietal cortex or in the frontal cortex according to others [55, 56].

Thirdly, a number of studies have demonstrated cognitive deficits in specific domains after brain tumor removal. For instance, some patients demonstrated minor deterioration in attention after resection of parenchymal frontal or precentral tumors [47, 57] and resection of the right prefrontal cortex rather than the left was associated with a selective attentional impairment in Stroop test performance [58]. After resection of the supplementary motor area, patients exhibited impaired procedural learning and agraphia [59, 60]. Subsets of patients with resections involving the frontal lobe demonstrated a variety of deficits. For instance, impaired sequence ordering of novel material was observed particularly in right-sided lesions, while recognition memory was unaffected [61], and planning and executive impairment, irrespective of side, site, and size [62, 63]. Furthermore, severe executive deficits in a reward learning task were observed in patients after bilateral fronto-orbital resections for various tumor types [64] and impaired virtual planning of real life activities after resections in the left and right prefrontal cortex, which could not be explained by memory deficits [65, 66].

In the year following surgery she noted more difficulty concentrating, and she was seizure free for 10 months, after which similar seizures reappeared, for which the carbamazepine was increased to 300 mg bid. After a year she divorced from her husband and started working as volunteer in a nursing home. Follow-up MRIs demonstrated slow increase of residual T2/FLAIR hyperintensity of approximately 4 mm per year. At 21 months her clinical condition is unchanged, but now there is a very extensive T2/FLAIR hyperintense infiltration and new multifocal gadolinium enhancement at the genu of the corpus callosum and left prefrontal area (Fig. 1e). Several options were considered: (1) radiotherapy and possibly adjuvant temozolomide at further progression, (2) new histopathology by either biopsy or limited resection, in case WHO grade 3 would be confirmed participation in a trial, in case WHO grade 4 would be confirmed chemo-irradiation. Because new histopathology would have implications for the radiotherapy plan, participation in a trial or concurrent temozolomide, a new histopathological diagnosis was advised by open biopsy, which would be methionine PET-guided towards the most anaplastic focus which was located prefrontally. The pathologist confirmed anaplastic oligoastrocytoma without necrosis, without 1p/19q loss, and with an extraordinarily high MIB1 labeling index. The rapid radiological progression in combination with the high labeling index were the arguments to start radiotherapy plus concomitant and adjuvant temozolomide at this point. She received 59.4 Gy using RapidArc technique. Again she developed more intense fatigue, lost 10 kg of weight, and developed a grade 4 thrombopenia and fever for which she was hospitalized for 2 weeks. MRI at 25 months, after the third cycle of adjuvant temozolomide, demonstrated a partial radiological response. Despite a combination of nausea, extreme fatigue, and anxiety for the future, she completed six cycles of temozolomide. She was unable to work and unable to care for her two children most of the time.

### Radiotherapy

Cognitive deficits are the hallmark of late-delayed encephalopathy [67], which is an irreversible and progressive complication that may follow radiotherapy by several months to many years through vascular injury causing ischemia of surrounding tissue and demyelination, local radionecrosis, and cerebral atrophy. The severity of cognitive deficits ranges from mild or moderate to dementia with progressive mental slowing and deficits in attention and memory, occurring in at least 12% of patients treated with radiotherapy [68]. In these cases, MRI shows diffuse atrophy with ventricular enlargement as well as severe confluent white-matter abnormalities [69]. There is a relation between cognitive status and cerebral atrophy and leukoencephalopathy [70, 71].

While short-term follow-up studies show limited or transient effects of radiotherapy [8], a number of studies in long-term survivors of low-grade glioma (i.e., more than 5 years following radiotherapy) concluded that radiotherapy in these patients poses a significant risk of long-term leukoencephalopathy and cognitive impairment. Surma-Aho et al. [72] reported low-grade glioma patients with a follow-up of 7 years to have more memory deficits after early radiotherapy than controls without radiotherapy. Moreover, leukoencephalopathy on MRI was more severe in the group with postoperative irradiation. A study among low-grade glioma survivors 6 years following diagnosis and initial treatment showed that the use of radiotherapy was associated with poor cognitive function on only a few tests and not restricted to one specific cognitive domain [73]. This finding suggests that cognitive deficits in these patients should not be attributed to radiotherapy, but rather to the tumor itself or other treatment factors, including epilepsy [74]. Serious memory deficits, however, are still to be expected when fraction doses exceed 2 Gy [73]. A recent follow-up of the Klein et al. [73] study demonstrated that regardless of fraction dose all tumor progression-free low-grade glioma patients that had irradiation showed a progressive deterioration in attentional functioning 13 years after radiotherapy while all patients without irradiation remained stable [70].

Again, the patient was seizure free for 10 months after chemoirradiation. When the seizures reappeared, she switched to levetiracetam 500 mg bid. Then she developed depressive feelings and lack of initiative for which she visited a psychologist. Because this was likely related to the levetiracetam, carbamazepine was reintroduced.

### Epilepsy and antiepileptic drug effects

The mechanism and pattern of seizures in brain tumor patients is determined by tumor type, tumor location and peritumoral and genetic changes in brain tumor patients [75]. Apart from tumor and treatment effects, cognitive function can be impaired by seizures [76]. An increased epilepsy burden has been found to adversely affect a broad range of cognitive functions [74] even to a larger extent than radiation therapy [73]. Decreased processing speed and attentional and executive deficits are notable sequelae of seizures and antiepileptic drugs (AEDs) in patients with brain tumors [73, 74, 77]. However, the literature is inconsistent on this point. Others did not detect any apparent effect of seizures on cognition across multiple cognitive domains assessed in a postsurgical sample [4]. Cognitive side-effects of AEDs can add to cognitive decline due to tumor effects, previous surgery, or radiotherapy, and therefore appropriate choice and dose of AED is crucial. The classical AEDs (phenytoin, carbamazepine, and valproic acid) are known to decrease cognitive functioning [78, 79]. Importantly, these drugs may also have pharmacological interactions with chemotherapy [80, 81] and thus potentially affect survival. These drugs may result in impaired attention and cognitive slowing, which can subsequently have effects on memory by reducing the efficiency of encoding and retrieval [79]. The importance of the classical AEDs as a risk factor for cognitive deficits has been reported in a study on stable disease, long-term low-grade glioma survivors [74] where reduced information processing speed, psychomotor function, working memory capacity, and executive functioning, were significantly related to the use of AEDs. As patients in this study who took AEDs had cognitive impairment even in the absence of seizures, the use of AEDs primarily affects cognitive function. Moreover, AED use in low-grade glioma patients may be associated with highly elevated levels of fatigue [82], which in itself is also associated with poorer cognitive outcome. Several new generation AEDs, like oxcarbazepine [83] and levetiracetam as add-on therapy [84], appear to have fewer adverse cognitive effects than the classical agents. Of the newer agents, topiramate is associated with the greatest risk of cognitive impairment, although this risk is decreased with slow titration and low target doses [85, 86]. It appears to be safe to switch patients from phenytoin to levetiracetam monotherapy following craniotomy for supratentorial glioma [87].

Follow-up MRIs of the patient every 3 months showed a stable response with no gadolinium enhancement and a T2/FLAIR hyperintense region that is progressive at 6 mm/year up to 44 months after initial surgery and 20 months after chemoirradiation. During this time she is still unable to work, and partly able to take care of her children due to concentration problems, fatigue, and depression.

A considerable number of brain tumor patients have feelings of anxiety, depression, and future uncertainty as psychological reactions to the disease [88–90]. These mood disturbances may lead to deficits in attention, vigilance, and motivation that subsequently affect several cognitive domains [91]. Loss of self confidence, unemployment, and dependency on caregivers may also negatively affect these patients’ cognitive status. Mood changes are more common in brain tumor patients than in patients with other neurological diseases [92] and might be related to tumor location [93]. Unilateral surgical removal of prefrontal cortex, including the fronto-orbital or anterior cingulate cortex, has resulted in emotional dysregulation with impaired voice and face expression identification in patients with various brain lesions including brain tumors [94]. Furthermore, deficits in recognizing emotional facial expression were observed after surgical removal of brain tumors that involved both heteromodal and limbic/paralimbic cortices [95]. Concordantly, impairment of arousal and emotional valence was demonstrated after resective surgery in various brain regions, but particularly in the right temporoparietal region [96]. This emotional impairment can have an impact on social and professional performance. Negative mood changes were observed after brain tumor resection involving heteromodal cortices located either prefrontal or temporoparietal, whereas positive mood changes were observed after lateral frontal resections [97]. Mood states did not correlate with laterality of the resection, tumor grading or lesion size.

At 44 months after initial surgery and 21 months after chemoirradiation, she experienced headache, a severe decline in cognitive functions, lethargia, and gait instability without seizures. At neurological examination she demonstrated bradyphrenia, disorientation, and a right-sided hemiparesis. She was admitted and a new MRI showed clear and sudden progression with extensive T2/FLAIR infiltration, and new gadolinium enhancement at the corpus of the corpus callosum and in the left internal capsula (Fig. 1). Because of her poor clinical condition and the extent of radiological progression, no further treatment options were considered and she received dexamethasone. She was discharged to a hospice and died at 46 months after initial surgical treatment.

### Corticosteroids

The potentially neurotoxic effects of corticosteroids are often misdiagnosed and underestimated [98] and corticosteroids may induce behavioral, psychic, and cognitive disturbances, due to functional and, over time, structural alterations in specific brain target areas. Corticosteroids may cause mood disturbances, psychosis, and cognitive deficits particularly in declarative memory performance. Steroid dementia is a reversible cause of cognitive deficits even in the absence of psychosis. Recent data suggest that transiently impaired attention, concentration, and memory are due to neurotoxic effects on both the hippocampal and the prefrontal areas [99]. Both short-term and long-term use of steroids has been associated with hippocampal-dependent explicit memory deficits [100]. More likely, however, corticosteroids may improve cognitive deficits because of resolution of edema [101].

## Relevance of neuropsychological testing

Comprehensive assessment of neurocognitive function is evidently different from the use of standard HRQOL measures, which is considered to be relatively easy and not time-consuming. However, while cognitive deterioration can be predictive of radiologic disease progression in patients with tumor recurrence [102], measures of HRQOL and activities of daily living are only weakly correlated to cognitive decline or to time to tumor progression, suggesting that HRQOL measures may not be sensitive enough to detect a change in patient function [102]. It should be noted, on the other hand, that HRQOL is an important outcome measure within the context of patient care [102]. Self-reported HRQOL measures or other methods of informal assessment of cognitive function depend on the patient’s report of their cognitive symptoms. Self-reports of decreased cognitive functioning au lieu of formal neuropsychological testing not only usually point at feelings of anxiety, depression, or fatigue rather than cognitive deficits [103, 104], and use of self-reports is even more problematic in brain tumor patients whose judgment may be severely impaired by the tumor [73].

## Surgical treatment considerations

Historically, when a low-grade glioma was diagnosed in a young, healthy adult, a commonly accepted strategy was a “wait and see” policy because of the presumed indolent nature and variable behavior of these tumors. However, retrospective studies of the kinetics of glioma growth, showed linear growth before anaplastic transformation [105]. The majority of low-grade gliomas are now known to progress to malignant gliomas with time. A better understanding of the natural history of low-grade gliomas has led to an interest in early treatment. The decision as to whether a patient with low-grade glioma should receive resection, radiotherapy, or chemotherapy is based on a number of factors including age, performance status, location of tumor, and patient preference. Since low-grade gliomas are such a heterogeneous group of tumors with variable natural histories, the risks and benefits of each of the three therapies must be carefully balanced with the data available from limited prospective studies. Since patients with low-grade gliomas can survive in a clinically stable state for several years after diagnosis, the long-term effects of the disease and its treatment on cognitive functioning of these long-term survivors are especially salient. Although not Class I evidence, numerous studies strongly suggest that more extensive surgical tumor resection is associated with longer life expectancy for both low- and high-grade gliomas [106]. At the same time, the rapid development of operative techniques and technologies, including brain mapping techniques aimed at preserving eloquent brain functions [107], facilitates the attainment of maximal or radiologically complete tumor resection while minimizing morbidity. Recently, supratotal resection extending beyond the radiological boundaries of the tumor, has been postulated to improve both survival and perseveration of function [108].

## Conclusions and assessment recommendations for neurocognitive testing

Cognitive functioning of brain tumor patients is an increasingly important outcome measure, because cognitive impairments can have a large impact on self-care, social and professional functioning, and consequently on HRQOL. Many factors contribute to cognitive outcome, such as direct and indirect tumor effects, seizures, medication, and oncological treatment. Review of the literature indicates that neurocognitive outcome in patients with primary brain tumors was assessed systematically in only a limited number of studies, and most involved a relatively small number of patients. Due to the absence of pretreatment neurocognitive assessments and treatment randomization the ability to differentiate between tumor effects and treatment effects, including surgery, was limited in the retrospective studies. However, studies among long-term low-grade glioma survivors indicated greater impairments in virtually all neurocognitive domains in patients who had radiotherapy compared to those who had not. Although the role of radiotherapy—for a long time thought to be the main cause of cognitive deficits in patients with brain tumors—has been studied extensively, the adverse effects on cognitive function of other tumor and treatment-related factors remain elusive. As far as resective surgery is concerned, both cognitive improvement and decline have been observed depending on pathology, lesion size, localization and laterality. Neurocognitive deficits, if present, are transient in most cases, except for low-grade glioma patients with tumors in the left hemisphere. AEDs may result in impairments of attention and neurocognitive slowing, which can subsequently have effects on memory by reducing the efficiency of encoding and retrieval. Cognitive outcome after resective surgery for brain tumors has not been systematically determined. Likewise, intrasurgical cognitive mapping to improve cognitive outcome also has not been systematically applied in these patients. Concerted action into studying the costs and benefits of presurgical, intrasurgical, and postsurgical cognitive assessments related to outcome of these patients is thus warranted.

Since a combination of cortical and subcortical lesions, epilepsy, surgery, radiotherapy, AEDs, corticosteroids, and psychological distress is likely to contribute to neurocognitive dysfunctioning in an individually unpredictable way, it would be most pragmatic to choose a core testing battery that gauges a broad range of neurocognitive functions. Additionally, the neuropsychological measures have to meet the following criteria: (i) assess several domains found to be most sensitive to tumor and treatment effects; (ii) have standardized materials and administration procedures; (iii) have published normative data; (iv) have moderate to high test–retest reliability; (v) have alternate forms or are relatively insensitive to practice effects, and are therefore suitable to monitor changes in neurocognitive function over time; (vi) include tests that have been translated into several languages (i.e., Dutch, English, French, German, Hebrew, Italian, Turkish) [109] or require translation primarily of test directions; and (vii) total administration time is 30–40 min. The neurocognitive domains deemed essential to be evaluated include attention, executive functions, verbal memory, and motor speed.

The test battery that meets most of the afore-mentioned criteria has successfully been used and is still being used in a number of EORTC, NCCTG, NCI-C, RTOG, MRC, and HUB multisite clinical trials and it has been shown that neurocognitive functioning has independent prognostic significance in patients with low-grade glioma [110]. Moreover, neurocognitive deterioration indicates tumor progression before signs of disease recurrence are evident on CT or MRI [3, 102, 111]. This battery assesses: *memory*, Hopkins verbal learning test [112], which is a list of 12 words in 3 semantic categories that measures immediate recall across 3 trials, recognition of the words from distractors, and delayed recall; *verbal fluency*, controlled oral word association [113], which requires the production of words beginning over a specific letter for three 1-min trials; *visual*-*motor scanning speed*, trail making test part A [114], which requires the subject to connect dots in numerical order as rapidly as possible; *executive function*, trail making test part B [114], which requires the subject to connect dots with alternating numbers and letters as rapidly as possible.
